# Patients with femoral or distal forearm fracture in Germany: a prospective observational study on health care situation and outcome

**DOI:** 10.1186/1471-2458-6-87

**Published:** 2006-04-04

**Authors:** Heinz G Endres, Burkhard Dasch, Margitta Lungenhausen, Christoph Maier, Rüdiger Smektala, Hans J Trampisch, Ludger Pientka

**Affiliations:** 1 Department of Medical Informatics, Statistics and Epidemiology, Ruhr University Bochum, D-44801 Bochum, Germany; 2 Department of Pain Management, BG-Kliniken Bergmannsheil, Ruhr University Bochum, D-44789 Bochum, Germany; 3 Department of Surgery, Knappschaftskrankenhaus Bochum-Langendreer, Ruhr University Bochum, D-44892 Bochum, Germany; 4 Department of Geriatrics, Ruhr University Bochum, Marienhospital Herne, D-44627 Herne, Germany

## Abstract

**Background:**

Distal radius and proximal femoral fractures are typical injuries in later life, predominantly due to simple falls, but modulated by other relevant factors such as osteoporosis. Fracture incidence rates rise with age. Because of the growing proportion of elderly people in Western industrialized societies, the number of these fractures can be expected to increase further in the coming years, and with it the burden on healthcare resources. Our study therefore assessed the effects of these injuries on the health status of older people over time. The purpose of this paper is to describe the study method, clinical parameters of fracture patients during hospitalization, mortality up to one and a half years after discharge in relation to various factors such as type of fracture, and to describe changes in mobility and living situation.

**Methods:**

Data were collected from all consecutive patients (no age limit) admitted to 423 hospitals throughout Germany with distal radius or femoral fractures (57% acute-care, femoral and forearm fractures; 43% rehabilitation, femoral fractures only) between January 2002 and September 2003. Polytrauma and coma patients were excluded. Demographic characteristics, exact fracture location, mobility and living situation, clinical and laboratory parameters were examined. Current health status was assessed in telephone interviews conducted on average 6–7 months after discharge. Where telephone contact could not be established, at least survival status (living/deceased/date of death) was determined.

**Results:**

The study population consisted of 12,520 femoral fracture patients (86.8% hip fractures), average age 77.5 years, 76.5% female, and 2,031 forearm fracture patients, average age 67.6 years, 81.6% female. Women's average age was 6.6 (femoral fracture) to 10 years (forearm fracture) older than men's (p < 0.0001). Only 4.6% of femoral fracture patients experienced changes in their living situation post-discharge (53% because of the fracture event), although less than half of subjects who were able to walk without assistive devices prior to the fracture event (76.7%) could still do so at time of interview (34.9%). At time of interview, 1.5% of subjects were bed-ridden (0.2% before fracture). Forearm fracture patients reported no change in living situation at all. Of the femoral fracture patients 119 (0.95%), and of the forearm fracture patients 3 (0.15%) died during hospital stay. Post-discharge (follow-up one and a half years) 1,463 femoral fracture patients died (19.2% acute-care patients, 8.5% rehabilitation patients), but only 60 forearm fracture patients (3.0%). Ninety percent of femoral fracture deaths happened within the first year, approximately 66% within the first 6 months. More acute-care patients with a pertrochanteric fracture died within one year post-discharge (20.6%) than patients with a cervical fracture (16.1%).

**Conclusion:**

Mortality after proximal femoral fracture is still alarmingly high and highest after pertrochanteric fracture. Although at time of interview more than half of femoral fracture patients reported reduced mobility, most patients (96%) attempt to live at home. Since forearm fracture patients were on average 10 years younger than femoral fracture patients, forearm fractures may be a means of diagnosing an increased risk of later hip fractures.

## Background

Both distal radius fractures and proximal femoral fractures are typical traumatic injuries affecting primarily elderly women. Women make up at least 70% of all patients with a distal radius fracture [1] or proximal femoral fracture [2]. More than 90% of these fractures occur in persons over 50 years of age.

The incidence of proximal femoral fracture for the year 1990 has been estimated at 1.3 to 1.7 million cases worldwide [3]. More than half of the cases occurred in the Western industrialized nations. In these countries, proximal femoral fractures are related to 1.1% of all deaths [3]. The incidence varies considerably from one country and region to another [4-6]. In Germany 110–130 cases per 100,000 inhabitants occurred in 2000 [7], with significant regional differences [8].

Even more frequent than hip fractures are fractures of the distal forearm, which account for up to 25% of all bone injuries in trauma surgery wards [5,9,10]. The incidence of distal forearm fractures in industrialized countries has been estimated at 200–300 per 100,000 inhabitants and is expected to increase further as the population ages [9,10]. Distal forearm fractures are seen by a number of authors as an indication of an increased risk of fractures of all kinds [11,12].

In older patients, the consequences of traumatic injuries are often very serious. Many fracture locations are associated with increased mortality in the first year after the fracture event [13,14]. This is particularly true of hip fractures [15-17]. Often the ability of the individuals affected to perform the activities of daily living and self-care is considerably reduced as well. Many patients are left with persistent mobility deficits, even those who were very active prior to the fracture event [18,19].

Because of the demographic shift in the Western industrialized nations toward a society with a growing proportion of elderly people, the number of hip fractures and distal forearm fractures can be expected to increase further in the coming years. This trend will have a major influence on the structure and costs of the health-care sector. For this reason, it is important to study the effects of these injuries on the health status of older people over time in a systematic way.

The aim of our prospective observational study was therefore to document clinical parameters of femoral fracture and distal forearm fracture patients during their stay in acute-care or rehabilitation hospitals, number of deaths up to one and a half years after discharge, and changes in mobility and living situation. This paper describes the methods and initial results of the study.

## Methods

### Subjects and facilities

Data were collected between January 2002 and September 2003 from all individuals (no age limit) admitted to one of the 423 participating hospitals with a femoral or distal forearm fracture. Polytrauma and coma patients were excluded. The hospitals, 242 acute-care facilities (both fracture types), 195 rehabilitation facilities (femoral fractures only), and 26 facilities with both acute-care and rehabilitation departments, were spread across all regions of Germany (urban as well as rural). Therefore the patient population in our study is broadly representative of the population of hospital patients in Germany with regard to age, sex and fracture location. The size of the participating facilities ranged from 0 to 2500 beds (median acute-care 314, median rehabilitation facilities 203). The recruitment and research protocols were reviewed and approved by the ethics committee of the Bavarian state medical association and the trial was undertaken in accordance with the Declaration of Helsinki. All subjects gave written, informed consent.

### Collection of data

Clinical data were collected into Case Report Forms (CRFs), entered by a commercial Contract Research Organization (CRO) into an Access™ database and sent to the central Oracle™ database at the University of Bochum. The data included: demographic details (age, sex, place of residence), living situation (home, nursing institution), exact anatomical fracture site (including accompanying fractures), treatment of fracture (surgical or non-surgical), ASA classification, clinical and laboratory parameters, bone density measurements as stated in the case history, mobility prior to fracture and at discharge (ranging from ambulatory without assistive devices to bed-ridden), drug treatment, and all deaths with date of death.

Subjects who had agreed to take part in a post-discharge telephone interview (conducted by a centralized call center at the University of Bochum) were contacted on average 6–7 months after discharge. The telephone interviews used evaluated questionnaires integrated into the interview software and available as computer screen masks (Oracle™ Forms 6.0 as graphic front-end). The software also managed the timely generating of patient contacts and interview administration. The interview process was completely paperless. All of the data collected were entered directly into the corresponding screen mask (electronic data capture). In this way the typical inputting errors associated with the change from one medium to another (paper to electronic data base) were avoided. Analysis of the data is not yet complete and will be reported in a subsequent publication. The interviewers also queried changes in mobility and living situation, as well as economic parameters such as medications (including OTC) or therapeutic measures such as physiotherapy. The average interview length was not to exceed 20 minutes, so as to prevent response bias as a result of overtaxing the respondent's ability to concentrate. The interviews were therefore designed in such a way that dichotomous decision criteria at various points determined the nature and number of questions that followed. Automatically guided by the Oracle software, blocks of questions could be omitted entirely. Questions that were potentially more difficult to answer were placed toward the end of the interview so as not to tire subjects excessively early on in the interview. For verbal rating scales the range of answers was limited to a maximum of 5 response categories per question. For each item, the category "don't know/no response" was available in case the patient was unable to understand or answer the question.

In the case of cognitively impaired subjects or subjects in poor health, a care-giving family member was interviewed if possible. In this case the self-rating questions were modified correspondingly. The caregiver rating option was offered only to persons in the femoral fracture group, since we could assume based on data in the literature that patients with forearm fracture would be considerably younger on average, and would therefore be capable of answering for themselves.

### Outcomes and statistical analyses

The most important outcome parameters were mortality and loss of independence and mobility. Extensive research was carried out to ensure that every death after discharge was recorded: first family members, where possible, then the participating physicians, and finally the local registry office of the particular individual were contacted. In this way it was possible to ascertain the status of 14,189 patients (98.3% of all) discharged from hospital. In addition to a univariate data analysis, possible significant differences between subgroups were tested. All analyses were carried out using SAS™ Statistical Software (Release 8.02, SAS Institute Inc., Cary, NC, USA).

## Results

### Subject characteristics

**Figure 1:**
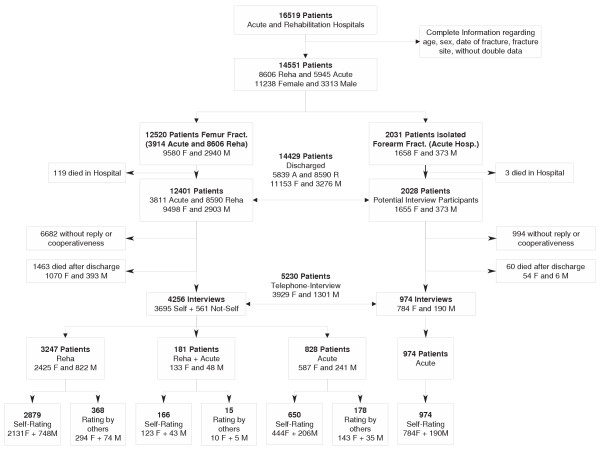
Study flow diagram.

**Figure 2:**
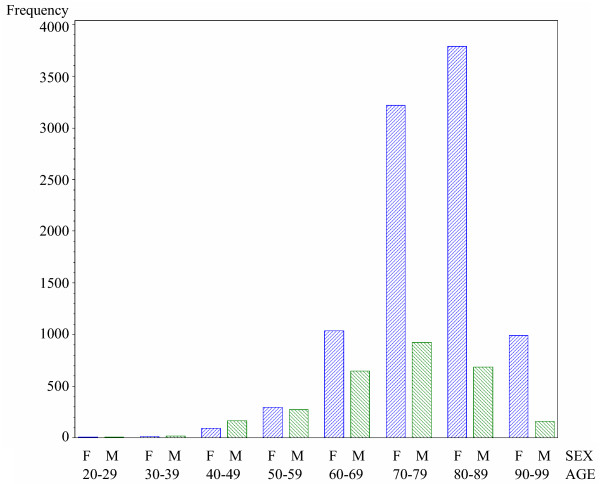
Age distribution of all 12,401 patients with femoral fracture at discharge.

**Figure 3:**
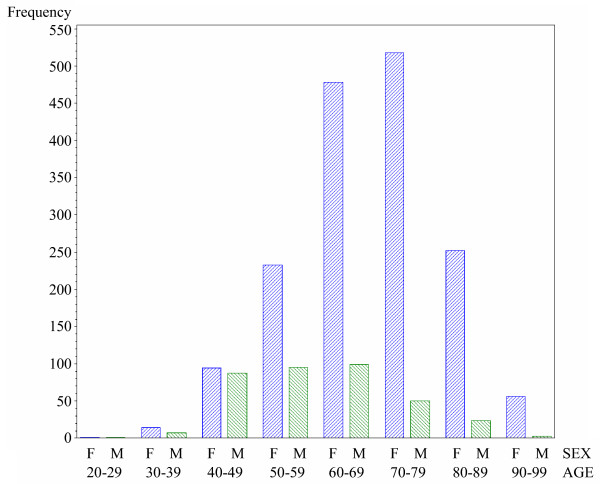
Age distribution of all 2,028 patients with distal forearm fracture at discharge.

16,519 patients were recruited, approximately 40% at acute-care facilities and 60% at rehabilitation facilities (Figure 1). After verification for data completeness, 14,551 (88.1%) patients remained (Table 1), 2031 with a distal forearm fracture – usually a fracture of the distal radius (81.6% women, on average 10 years older than men) – and 12,520 with a femoral fracture (76.5% women, on average 6.6 years older than men), of which 86.8% were proximal. Patients with distal forearm fracture were younger (10 years on average, Figure 2 and 3), had significantly fewer comorbidities and significantly better ASA classifications (over 80% ASA grades I-II). There was also a significant difference between femoral and forearm fracture patients with regard to BMI (p < 0.0001): the forearm fracture group included the larger proportion of obese patients (15% with a BMI ≥ 30 compared to 11% of femoral fracture patients).

### Mortality rates

During hospital stay, 103 (2.6%) acute-care and 16 (0.2%) rehabilitation patients with a femoral fracture died, and 3 (0.1%) acute-care patients with a distal forearm fracture did so (Figure 1). Within one and a half years post-discharge 1,463 femoral fracture patients (11.8% of all) had died: 19.2% of all acute-care patients, but only 8.5% of all rehabilitation patients (Table 2). Ninety percent of these deaths happened within the first year, approximately 66% within the first 6 months. In contrast, only 60 forearm fracture patients (3.0%) died within one and a half years of discharge. In terms of the two most common femoral fracture locations (83.8% of all femoral fractures), mortality rate within the first year (Table 3) was significantly higher among patients with pertrochanteric fracture (mortality 13.2% of all, 20.6% of acute-care patients), than among those with cervical fracture (mortality 9.8% of all, 16.1% of acute-care patients). This applies to both types of hospital but not to sex; a significant difference between the two locations was found only with women.

### Fracture site, operative treatment, and previous fractures

The two most common femoral fracture locations reported were the cervical (45.7%) and the pertrochanteric fracture (38.1%), which together with subtrochanteric fractures (2.9%) make up the group of proximal femoral fractures (Table 4). The remaining 13.3% of femoral fracture patients presented with distal femoral fractures, combination fractures, or unclassified. No lateral predominance was observed in either sex in femoral fractures (right and left hip near 50%) in contrast to distal forearm fractures which occurred significantly more frequently on the left side in women (left arm 58.9%, right arm 39.6%, both arms 1.5%) but not in men (left arm 48.4%, right arm 50%, both arms 1.6%). Close to 100% of patients were operated on for most fracture sites. The most frequent procedures were nailing, THP, and bipolar femoral head prosthesis (Table 5).

Thirty-two percent of all femoral fracture patients and 28% of all distal forearm fracture patients had sustained previous fractures (Table 1), most frequently radius fractures (11.2%–12.6%), followed by fractures of the femur (3.0%–7.4%). Low bone density was reported for 25.9% of acute-care femoral fracture patients and 37.0% of rehabilitation patients. The significant difference is most probably due to better possibilities for measuring low bone density in rehabilitation facilities. When acute-care patients with femoral and distal forearm fractures are compared directly, the percentages with low bone density are nearly identical. For most previous fracture locations, the frequency in femoral fracture patients was about twice as high as in forearm fracture patients, most likely due to the fact that forearm fracture patients were on average 10 years younger.

### Telephone interviews

At the end of their hospital stay, all patients were asked whether they would be willing to take part in a telephone interview 6 months to a year later. Of the 12,401 patients with a femoral fracture, 10,964 (88.4%) consented, and of the 2,028 forearm fracture patients 1,752 (86.4%) did so. Of the femoral fracture patients who consented to interviews, 1,184 (10.8%) died after discharge (all deaths after discharge: 11.8%; see Table 2). Of the forearm fracture patients willing to be interviewed 40 (2.3%) died post-discharge (all deaths after discharge: 3.0%; see Table 2).

This left a total of 9,780 (78.9%) femoral fracture patients and 1,712 (84.4%) forearm fracture patients as potential interview participants. Attempts were made to contact all of these patients, and ultimately there were only 56 (0.6%) femoral fracture patients and 6 (0.4%) forearm fracture patients with whom no telephone contact could be established. With all other patients or their caregivers, generally family members, we were able to establish at least a first telephone contact. At the time of this first contact, 5,468 (55.9%) femoral fracture patients and 732 (42.8%) forearm fracture patients withdrew their consent to be interviewed. In the end, we were able to interview 43.5% (femur) and 56.9% (forearm) of the potential interview participants. Interviews were conducted with these 5,230 patients on average 6–7 months post-discharge (183 ± 58 days in the case of femoral fracture patients and 221 ± 58 days in the case of distal forearm fracture patients).

Self-rating telephone interviews could be conducted with 86.8% (3695) of the femoral fracture patients, while the remaining interviews (13.2%) were conducted with caregivers (Figure 1). We intentionally chose to conduct interviews with caregivers where necessary, in order to avoid poor health becoming a selection criterion for an interview. Other characteristics, however, inevitably act as selection criteria for a telephone interview, as recently described [20]. It is known, for example, that interview participants tend to be significantly younger on average than non-participants. This was true in the present study except for men with forearm fracture, although the age difference was small (4–6 years) and therefore negligible (Table 6). It is also known [20] that men are more likely to consent to be interviewed than women, although in our study this difference was found only among femoral fracture patients. Interview participants also had lower ASA classifications overall (with the exception of men with forearm fracture), but at 0.3 points this difference is so small as to be clinically irrelevant, and it was only because of the large number of patients that it is nevertheless statistically significant (Table 6). Comparing the data on mobility and living situation of femoral fracture patients in acute-care and rehabilitation facilities, one finds that patients who participated in an interview were on average more mobile and more independent than those with whom no interview was conducted (p < 0.0001).

### Changes in mobility in patients with femoral fracture

Prior to the fracture event the majority of femoral fracture interview patients were mobile without assistive devices (76.7% of all, 71.7% of acute-care patients, and 80.2% of rehabilitation patients). One-fifth needed assistive devices (19.6% of all, 24.8% of acute-care patients, and 18.9% of rehabilitation patients) and only a minority were bedridden (0.7% and 0.2% respectively) or required the help of assistive persons (2.8% and 0.7% respectively). By the time of the interview the situation had changed completely: only 34.9% of all (31.0% of acute-care patients, 36.4% of rehabilitation patients) were still mobile without assistive devices. The majority now needed such devices (62.0% of all, 64.1% of acute-care patients, 61.5% of rehabilitation patients). Yet the living situation of the interview patients had changed very little: 97.4% lived at home prior to fracture and 96.3% still did so after discharge. Only 4.6% experienced changes in their living situation post-discharge (53% because of the fracture event) and a small minority of 3.0% of all femoral fracture patients who were interviewed stated that they had moved to a nursing home.

## Discussion

In this large study, we had the opportunity to collect data on all patients with a femoral fracture or a fracture of the distal forearm admitted to 423 hospitals over a 21-month period ending in September 2003. The 14,551 study participants are broadly representative of patients with these types of fractures in Germany with regard to age, sex, fracture location, mobility prior to the fracture event, and comorbidities. Women made up almost 77% of all femoral fracture patients and 82% of all patients with a fracture of the distal forearm. The majority were post-menopausal, similar to the results of previous studies [1,5,6,21]. However, in contrast to recently published results from England and Wales [5], fractures of the femur outnumbered forearm fractures. The most probable reason for this lies in the way the data were collected. Whereas the data in the British study were collected from family doctors in private practice (General Practice Research Database), the data in our study consisted of patients admitted to hospital. Therefore, from the fact that in our study femoral fractures outnumbered forearm fractures, one can deduce that in Germany forearm fracture patients are treated in non-hospital settings as well.

Osteoporosis is a known risk factor for the occurrence of fractures [11,22]. Both distal forearm fractures and femoral fractures are linked to osteoporosis, but the effect on forearm fractures occurs at an earlier age [23]. This is reflected in our study, where one fourth of all acute-care patients had reduced bone density. Since the forearm fracture patients were on average 10 years younger than the femoral fracture patients, one can assume that the bone density is already markedly reduced at a younger age. Therefore, in accordance with the results of our study, forearm fractures (the most frequent prior fractures) may be a means of diagnosing an increased risk of later hip fractures [11,12,22,24]. The predominance of left forearm fractures in women observed in our study is also most probably due to lower bone density in the non-dominant forearm (usually the left) [1]. In men, differences in bone density are less pronounced and hence no significant lateral difference was observed.

Another well known risk factor for fractures, and beyond that for poorer treatment outcomes, is cognitive impairment [25,26]. Moreover, cognitive impairment makes it difficult if not impossible to obtain interview data from a patient. Other studies have therefore abstained from interviewing these patients, since it was assumed either that it would be impossible to question them [27], or that their condition would have a confounding effect on the responses [15]. One of the strengths of our study is that it did not exclude cognitively impaired patients. Excluding data for such patients would inevitably lead to an overestimation of the quality of treatment outcomes. We solved this problem by using a second version of the questionnaire developed exclusively for persons caring for these patients, so as to collect outcome data on all patients at time of interview. In this way we succeeded in obtaining interview data from an additional 561 patients (13.2%), who suffered from cognitive impairment (447 women and 114 men).

Studies in the last three years have presented a wide range of one-year mortality rates after hip fracture, ranging from 11.4% [27-29] to 24% [14,30,31], with a higher rate for men compared to women [5,25,29,32,33], and a mortality in the following years that is significantly less, though still higher than in the general population [13]. In contrast to hip fractures, the one-year mortality rate after forearm fracture is only 2–6%, which does not differ significantly from the mortality rate in the population as a whole [5,13,14]. Known risk factors for mortality after fractures are underweight and male sex [14,32,34,35]. Many publications have linked male sex to a two- to fourfold rise in mortality [32,34].

Our study gives a more detailed picture of the mortality rate. We identified differences in mortality by type of facility, fracture site, age, sex, and health status. More than twice as many acute-care femoral fracture patients as rehabilitation patients died within one and a half years post discharge (19.2% versus 8.5%). This reflects the fact that acute-care patients with a poor health status are not transferred to rehabilitation facilities, as evidenced by the more favorable ASA classification of rehabilitation patients, 66% of whom were classified as ASA grades I-II, compared to 39.5% ASA grades I-II for acute-care patients. The ASA classification as a predictor of long-term mortality was confirmed by another recent study [27]. In contrast to a previous publication [36], we can also demonstrate an association between the number of medical comorbidities, which is significantly higher in acute-care femoral fracture patients (Table 1), and an increased one-year mortality rate in acute-care patients, which is no surprise considering that multi-morbidity accompanies poorer ASA rating. We also found significant differences in BMI values: more femoral fracture patients had a BMI < 25, which can be seen as an indication of poor general status in significantly older patients, and more acute-care femoral fracture patients had a BMI < 25, which corresponded with their poorer ASA classification. The predictive value of nutritional status on outcomes for fracture patients was described recently [14,35].

Ninety percent of post-discharge deaths happened within the first year, approximately 66% within the first 6 months. This is consistent with White's observation [37] that the effects of hip fracture on mortality are seen primarily in the first year after fracture. The one-year mortality rate for men and women is similar to those given in recent publications with mortality rates in the upper range [13,14,30,37]. Although significantly more men than women died, men in our study were on average 5 years younger than women at time of death. Assessment of mortality in relation to the two most common femoral fracture sites (Table 3) shows that patients at acute-care facilities with pertrochanteric fracture had a one-year mortality of almost 21%, while those with a cervical fracture had a one-year mortality of only 16%, a clear difference in mortality between the two fracture locations that was also found by Thorngren [33] at 4 months post-fracture (pertrochanteric fracture 14.0% mortality, cervical hip fracture 11.9% mortality).

Contrary to the assumptions in a previous publication [28] this finding cannot be explained by the very small differences in age or ASA classification (Table 3). Other reasons must be taken into consideration such as differences in postoperative complications due to different operative procedures (nailing or screws in 96% of pertrochanteric fractures, THP or bipolar femoral head prosthesis in 81% of cervical fractures) or surgery delays [38]. Among the rehabilitation patients too, pertrochanteric fracture and cervical fracture were the two most common fracture sites, but the difference in one-year mortality rate is much smaller (9.3% pertrochanteric fracture versus 7.0% cervical fracture). The most probable explanation is again the better health status of the average rehabilitation patient. The better health status of the younger forearm fracture patients (one decade on average), as reflected in a significantly lower incidence of comorbid diagnoses and a significantly better ASA classification, is also the most obvious reason for their low mortality rates (3.3% of all women, 1.6% of all men, Table 2), which does not differ significantly from the mortality rate in the population as a whole [5,13,14].

Mobility and the ability to perform basic activities of daily living without help are essential to patients' functional independence. In keeping with earlier studies [17,19,34,39], we found that hip fractures, but not forearm fractures, had a clear negative effect on those abilities, with less than half as many patients mobile without assistive devices at time of interview compared to the situation prior to fracture event. But, even though we can demonstrate a distinct loss in mobility, the percentage of interview patients who lived at home prior to the fracture event (97.4%), and the percentage who were discharged to home after their stay in rehabilitation hospitals (96.3%), is nearly identical. The small percentage of patients (3%) who reported at the interview that they lived in a nursing home does not match the substantially higher rates found in other studies [6,25,40-42]. Cumming [41] for example found that 27% of the cases and 5% of controls who lived at home were in a nursing institution one year later. In the study by Marottoli [25] the rate was 23% after six months. Thorngren [6] reported that in 2000 in Sweden only 50% of all hip fracture patients returned to their own home. However, only 62.7% of these patients lived in their own home before the fracture event, while 9.2% lived in nursing homes and the rest in institutional care. The differences between these results and our own may be due to the fact that places in nursing homes are expensive in Germany, and consequently patients attempt to assure ambulatory care at home. On the other hand the percentage of patients in our study who were able to walk without the help of assistive persons at time of interview was very high (95.1% acute-care patients and 97.9% rehab patients). So it seems that femoral fracture may only be a risk factor for long-term institutionalization if the subject's ability to independently perform activities of daily living is substantially reduced (requiring the help of assistive persons or bedridden).

Our study has several evident strengths: the very large number of participants, the efforts to obtain important outcome parameters of cognitively impaired patients, and the extensive research carried out to determine the survival status of almost 100% of all discharged patients. Nevertheless, the results should be interpreted in the context of several limitations. Since the interviews were not designed to obtain information from doctors or family members about the mobility or living situation of patients who had died in the meantime, it is possible that the need for assistance and the proportion of patients who were institutionalized after discharge from the hospital has been underestimated. The possibility of erroneous responses by patients as well as caregivers also cannot be ruled out. Furthermore our prospective study did not include a control group, so that we can not compare the marked loss of mobility with a potential analogous loss of mobility in controls.

## Conclusion

Women made up the majority (77%–82%) of all hospital patients with the assessed fracture types. Most were post-menopausal and the mean age was 6.6–10 years older than that of men. The role of osteoporosis as a contributing factor in fractures is seen in the incidence of reduced bone density in both fracture types and a significant lateral difference in distal forearm fractures in women. Forearm fractures occurred on average one decade earlier than did femoral fractures (67.6 years versus 77.5 years) and could serve as an indicator for risk of subsequent fractures later in life. The mortality rate of acute-care femoral fracture patients within one and a half years after discharge was still alarmingly high at 19.2% (20% of all male and 19% of all female patients). Ninety percent of these deaths occurred within the first year, approximately 66% within the first 6 months. Especially at risk were patients with pertrochanteric fracture, who had a one-year mortality of almost 21%. This knowledge identifies a subpopulation of hip fracture patients who may benefit from closer monitoring especially during the postoperative period. Femoral fractures also had a clear negative effect on mobility and the ability to perform basic activities of daily living, with less than half as many patients mobile without assistive devices at time of interview compared to the situation prior to fracture event. Nevertheless, the percentage of patients that lived at home prior to fracture event and after rehabilitation remained nearly identical. It would appear that patients seek the help they need to allow them to continue living at home as long as possible. This has important implications for the patient's families and for the health-care system, since the increased need for home care will entail an expansion of home-care services.

## Abbreviations

CRF: Case Report Form

CRO: Contract Research Organization

ASA: American Society of Anesthesiologists physical status risk rating

ADL: Activities of Daily Living

GPA: Global Patient Assessment

GCPS: Graded Chronic Pain Scale (von Korff questionnaire)

OTC: Over the Counter Drugs

## Competing interests

The author(s) declare that they have no competing interests.

## Authors' contributions

**HGE **wrote the manuscript and participated in acquisition of data, data management, and statistical analysis and interpretation of data.

**BD **participated in data management, statistical analysis and interpretation of data, and critically revised the manuscript.

**ML **conducted most of the patient interviews, participated in questionnaire design and critically revised the manuscript.

**CM **contributed to the study design, was responsible for the questionnaires, and participated in the coordination of the study.

**RS **participated in writing the study protocol, was responsible for the interpretation of surgical data, and critically revised the manuscript.

**HJT **made substantial contributions to conception and design of the study, participated in writing the study protocol, and participated in study coordination and statistical analysis.

**LP **is principal investigator. He was partly responsible for the study design, participated in analysis and interpretation of data, and helped to draft the manuscript.

All authors read and approved the final manuscript.

## Pre-publication history

The pre-publication history for this paper can be accessed here:



## Figures and Tables

**Table 1:** Data for all 14,551 patients at time of admission by indication and type of hospital

	**Femoral fracture patients**			**Forearm fracture patients**
	**All hospitals**	**Acute-care hospitals**	**Rehabilitation hospitals**	**Acute-care hospitals**

**Number of patients**	**12,520**	**3914 (31.3%)**	**8606 (68.7%)**	**2031 (100%)**

**Sex**	F: 9580 (76.5%)	F: 2975 (76.0%)	F: 6605 (76.7%)	F: 1658 (81.6%)
	M: 2940 (23.5%)	M: 939 (24.0%)	M: 2001 (23.3%)	M: 373 (18.4%)

**Age: mean (± SD)**	F: 79.1 (9.9)	Comparison acute-care versus rehabilitation facilities:		F: 69.4 (12.3)
	M: 72.5 (12.9)	F: 81.3 (9.8) versus 78.1 (9.9) Δ = 3.2 years (p < 0.0001)		M: 59.4 (13.8)
	(p < 0.0001)	M: 73.8 (13.3) versus 71.9 (12.6) Δ = 1.9 years (p < 0.0002)		(p < 0.0001)

**BMI (kg/m^2^)**	< 25: 56.3%	Acute-care versus rehabilitation: < 25: 60.6% vs. 54.4%		< 25: 42.7%
	25–29.9: 32.6%	25–29.9: 30.8% vs. 33.4%		25–29.9: 42.2%
	≥ 30: 11.1%	≥ 30: 8.6% vs. 12.2%		≥ 30: 15.2%
		(p < 0.0001)		

**ASA classification**	(I) 11.1%	Acute-care versus rehabilitation: (I): 5.3% vs. 13.6%		(I) 28.8%
	(II) 46.7%	(II): 34.2% vs. 52.4%		(II) 51.4%
	(III) 38.7%	(III): 55.2% vs. 31.3%		(III) 19.1%
	(IV+V) 3.6%	(IV+V): 5.3% vs. 2.7%		(IV+V) 3.6%
		(p < 0.0001)		

**Presenting fracture (% of all patients)**	Proximal femur: 10,865 (86.8%) Distal femur: 138 (1.1%) Combination (usually with radius) or unclassified: 1,517 (12.1%)	Proximal femur: 3623 (92.6%) Distal femur: 27 (0.7%) Combination (usually with radius) or unclassified: 264 (6.7%)	Proximal femur: 7242 (84.2%) Distal femur: 111 (1.3%) Combination (usually with radius) or unclassified: 1253 (14.6%)	F: left arm 58.9%, right arm 39.6%, both arms 1.5% M: left arm 48.4%, right arm 50%, both arms 1.6%

**Previous fracture (% of all patients)**	4010 (32.0%)	1170 (29.9%)	2840 (33.0%)	569 (28.0%)

**Fracture location (% of all patients with a previous fracture, multiple locations possible)**	Radius: 11.2%	Radius: 10.4%	Radius: 11.6%	Radius: 12.6%
	Humerus: 4.5%	Humerus: 4.3%	Humerus: 4.7%	Humerus: 2.8%
	Vertebra: 4.4%	Vertebra: 3.7%	Vertebra: 4.7%	Vertebra: 2.6%
	Femur: 7.4%	Femur: 7.2%	Femur: 7.5%	Femur: 3.0%
	Other: 12.4%	Other: 11.2%	Other: 12.9%	Other: 11.8%

**Low bone density**	33.7%	25.9% of 792 patients	37.0% of 1889 patients	23.4% of 590 patients

**Reported comorbidities (% of all patients, multiple comorbidities possible)**	Hypertension: 61.2%	Hypertension: 64.6%	Hypertension: 59.6%	Hypertension: 48.9%
	Diabetes: 20.5%	Diabetes: 22.6%	Diabetes: 19.5%	Diabetes: 11.3%
	CHD: 36.0%	CHD: 45.1%	CHD: 31.7%	CHD: 21.2%
	MI: 7.0%	MI: 7.6%	MI: 6.7%	MI: 3.2%
	PAD: 13.5%	PAD: 20.7%	PAD: 10.2%	PAD: 8.2%
	Stroke: 11.8%	Stroke: 14.0%	Stroke: 10.9%	Stroke: 4.8
	Arthritis: 34.8%	Arthritis: 30.0%	Arthritis: 36.9%	Arthritis: 23.9%
	Rheumatism: 5.0%	Rheumatism: 5.4%	Rheumatism: 4.8%	Rheumatism: 3.6%
	COPD: 11.4%	COPD: 13.1%	COPD: 10.7%	COPD: 7.4%
	Malignant tumour: 10.1%	Malignant tumour: 10.0%	Malignant tumour: 10.2%	Malignant tumour: 5.9%
	Kidney dysfunction: 6.0%	Kidney dysfunction: 8.9%	Kidney dysfunction: 4.6%	Kidney dysfunction: 2.0%

ASA classification: I = normal healthy patient; II = patient with mild systemic disease; III = patient with severe systemic disease that is not incapacitating; IV = patient with incapacitating systemic disease that is a constant threat to life; V = moribund patient/CHD = Coronary Heart Disease/MI = Myocardial Infarction/PAD = Peripheral Arterial Disease/COPD = Chronic Obstructive Pulmonary Disease

**Table 2:** Mortality data during hospital stay and post discharge

	**Acute-care/femoral fracture**	**Rehabilitation/femoral fracture**	**Acute-care/forearm fracture**
**All admission patients**	**12,520**		**2031**
**Number of admission patients by type of hospital**	**3914 (31.3%) F: 2975 (76.0%)/M: 939 (24.0%)**	**8606 (68.7%) F: 6605 (76.7%)/M: 2001 (23.3%)**	**2031 (100%) F: 1658 (81.6%)/M: 373 (18.4%)**
**Deceased during hospital stay (mean age ± SD)**	103 (2.6% of 3914) 85.3 years (± 8.2)	16 (0.2% of 8606) 85.5 years (± 7.3)	3 (0.15% of 2031)
**Sex breakdown of deceased pa-tients: N and mean age (± SD)**	F: 72 (2.4% of 2975) 86.7 years (± 6.6) M: 31 (3.3% of 939) 81.9 years (± 10.4)	F: 10 (0.15% of 6605) 85.8 years (± 7.3) M: 6 (0.3% of 2001) 85.1 years (± 7.9)	F: 3 (0.2% of 1658) 81.3 years (± 6.6)
**t-Test age difference**	p = 0.005	p = 0.86	
**All discharge patients**	**12,401**		**2028**
**Number of discharged patients by type of hospital**	**3811 (30.7% of 12,401) F: 2903 (76.2%)/M: 908 (23.8%)**	**8590 (69.3% of 12,401) F: 6595 (76.8%)/M: 1995 (23.2%)**	**2028 F: 1655 (81.6%)/M: 373 (18.4%)**
**Deceased any time post discharge (mean age ± SD)**	733 (19.2% of 3811) 83.9 years (± 8.7)	730 (8.5% of 8590) 81.6 years (± 9.4)	60 (3.0% of 2028) 80.4 years (± 9.9)
**Sex breakdown deceased pa-tients: N and mean age (± SD)**	F: 551 (19.0% of 2903) 85.0 years (± 8.0) M: 182 (20.0% of 908) 80.6 years (± 9.9)	F: 519 (7.9% of 6595) 83.0 years (± 8.6) M: 211 (10.6% of 1995) 78.1 years (± 10.3)	F: 54 (3.3% of 1655) 81.2 years (± 9.3) M: 6 (1.6% of 373) 73.7 years (± 13.1)
**t-Test age difference deceased patients**	p < 0.0001	p < 0.0001	p = 0.08
**Chi-sq sex vs. hospital type deceased patients**	p = 0.08		

**Table 3:** Patients with pertrochanteric or cervical fracture who died in the first year post discharge: comparison of sex, age, ASA and BMI

**Fracture location**	**Patients deceased in the first year N (percentage),** ** or mean (± SD)**		
	**Acute-care**	**Rehabilitation**	
**Pertrochanteric: 625 **(13.2% of 4730)	335 (20.6% of 1624)	290 (9.3% of 3106)	Chi square acute-care: p = 0.0007 Chi square rehabilitation: p = 0.0003
**Cervical: 554 **(9.8% of 5662)	281 (16.1% of 1744)	273 (7.0% of 3918)	
			
	**Women**	**Men**	
**Pertrochanteric: 625**	473 (13.1% of 3603)	152 (13.5% of 1127)	Chi square women: p < 0.0001 Chi square men: P = 0.477
**Cervical: 554**	388 (9.0% of 4336)	166 (12.5% of 1326)	
			
	**Age Women**	**Age Men**	
**Pertrochanteric: 625**	85.1 (± 7.9)	78.6 (± 11.2)	t-Test women: p = 0.0006 t-Test men: p = 0.45
**Cervical: 554**	83.2 (± 8.0)	79.5 (± 9.4)	
			
	**ASA Women**	**ASA Men**	
**Pertrochanteric: 625**	2.8 (± 0.6)	2.8 (± 0.7)	t-Test women: p = 0.005 t-Test men: p = 0.98
**Cervical: 554**	2.7 (± 0.7)	2.8 (± 0.7)	
			
	**BMI Women**	**BMI Men**	
**Pertrochanteric: 625**	23.2 (± 4.5)	24.1 (± 4.0)	t-Test women: p = 0.26 t-Test men: p = 0.84
**Cervical: 554**	23.6 (± 4.6)	24.0 (± 3.8)	

**Table 4:** Patients with femoral fracture: reported fracture location at discharge, two locations possible

**Certain or most probable fracture location: Total (% of 12,401)**	**Acute-care facilities 3,811 patients**		**Rehabilitation facilities 8,590 patients**	
	**Total (% of 3,811)**	**Women**	**Total (% of 8,590)**	**Women**
**Cervical: 5662 (45.7%)**	1744 (45.8%)	1321 (75.7%)	3918 (45.6%)	3015 (77.0%)
**Pertrochanteric: 4730 (38.1%)**	1624 (42.6%)	1229 (75.7%)	3106 (36.2%)	2374 (76.4%)
**Subtrochanteric: 365 (2.9%)**	160 (4.2%)	119 (74.4%)	205 (2.4%)	155 (75.6%)
**Distal: 135 (1.1%)**	24 (0.6%)	19 (79%)	111 (1.3%)	85 (76.6%)
**Periprosthetic: 33 (0.3%)**	12 (0.3%)	8 (67%)	21 (0.2%)	16 (76%)
**Combination fracture: 676 (5.5%)**	224 (5.9%)	186 (83.0%)	452 (5.3%)	359 (79.4%)
**Unclassified fracture: 800 (6.5%)**	23 (0.6%)	21 (91%)	777 (9.0%)	591 (76.1%)

**Table 5:** Femoral fracture patients: Reported operative procedure by fracture location at discharge

	**Fracture location**					
**Acute-care patients at** ** discharge (n = 3811)**	**Cervical** ** (n = 1744)**	**Pertrochanteric** ** (n = 1624)**	**Subtrochanteric** ** (n = 160)**	**Distal** ** (n = 24)**	**Other, incl. peripros-thetic,** ** or unclassified (n = 35)**	**Combination** ** (n = 224)**

OP yes (%)	1673 (96.3%)	1612 (99.4%)	156 (97.5%)	23 (95.8%)	31 (88.6%)	217 (96.9%)
OP no (%)	65 (3.7%)	10 (0.6%)	4 (2.5%)	1 (4.2%)	4 (11.4%)	7 (3.1%)
Missing (n)	6	2	0	0	0	0
						
**Rehabilitation patients at discharge (n = 8590)**	**Cervical (n = 3918)**	**Pertrochanteric (n = 3106)**	**Subtrochanteric (n = 205)**	**Distal (n = 211)**	**Other, incl. peripros-thetic, or unclassified (n = 798)**	**Combination (n = 452)**
OP yes (%)	3355 (98.6%)	2652 (99.7%)	175 (100%)	101 (98.1%)	665 (97.2%)	374 (90.8%)
OP no (%)	46 (1.4%)	8 (0.3%)	0	2 (1.9%)	19 (2.8%)	38 (9.2%)
Missing (n)	517	446	30	108	114	40
						
**All femoral fracture patients at discharge**	**Cervical (n = 5662)**	**Pertrochanteric (n = 4730)**	**Subtrochanteric (n = 365)**	**Distal (n = 235)**	**Other, incl. peripros-thetic, or unclassified (n = 833)**	**Combination (n = 676)**
**Total number of OPs (acute and rehab)**	5028 (97.8%)	4264 (99.6%)	331 (98.8%)	124 (97.6%)	696 (96.8%)	591 (92.9%)
**Procedure (more than one response possible)**	**% each in reference to total number of known OPs**					
Nailing	237 (4.7%)	2833 (66.4%)	251 (75.9%)	57 (46.0%)	246 (35.3%)	227 (38.4%)
THP	2046 (40.7%)	105 (2.5%)	17 (5.1%)	11 (8.9%)	191 (27.4%)	105 (17.8%)
Screw or screws	943 (18.8%)	1280 (30.0%)	63 (19.0%)	19 (15.3%)	146 (21.0%)	137 (23.2%)
Intramedullary rod	14 (0.3%)	115 (2.7%)	8 (2.4%)	2 (1.6%)	18 (2.6%)	9 (1.5%)
Monopolar femoral head prosthesis	204 (4.1%)	12 (0.3%)	1 (0.3%)	2 (1.6%)	12 (1.7%)	13 (2.2%)
Bipolar femoral head prosthesis	2011 (40.0%)	72 (1.7%)	8 (2.4%)	2 (1.6%)	150 (21.6%)	111 (18.8%)
Other (incl. ligament suture)	82 (1.6%)	241 (5.7%)	15 (4.5%)	9 (7.3%)	33 (4.7%)	63 (10.7%)
K wire	7 (0.1%)	10 (0.2%)	2 (0.6%)	2 (1.6%)	7 (1.0%)	52 (8.8%)
Plate	112 (2.2%)	496 (11.6%)	53 (16.0%)	46 (37.1%)	71 (10.2%)	70 (11.8%)

**Table 6:** Patients at discharge and interview respondents: comparison of sex, age, and ASA

	**Femur**		**Distal Forearm**	
	**Discharge without interview participation afterwards**	**Discharge with interview participation afterwards**	**Discharge without interview participation afterwards**	**Discharge with interview participation afterwards**

**Number of patients**	8,145	4,256	1,054	974
**Number of patients Acute-care and rehab hospitals**	Acute F: 2279/M: 651	Acute F: 624/M: 257	Acute F: 871/M: 183	Acute F: 784/M: 190
	Rehab. F: 4074/M: 1141	Rehab. F: 2521/M: 854		
**Sex distribution (%) and Chi square**	F: 6353 (78.0%)	F: 3145 (73.9%)	F: 871 (82.6%)	F: 784 (80.5%)
	M: 1792 (22.0%)	M: 1111 (26.1%)	M: 183 (17.4%)	M: 190 (19.5%)
	p < 0.0001		p = 0.21	
**Age in years mean (± SD) t-Test**	F: 80.6 (± 9.4)	F: 75.7 (± 10.2)	F: 72.1 (± 12.7)	F: 66.4 (± 11.0)
	M: 73.8 (± 13.0)	M: 70.2 (± 12.4)	M: 59.7 (± 15.7)	M: 59.1 (± 11.8)
	p < 0.0001	p < 0.0001	p < 0.0001	p < 0.0001
**Δ Age [95% CI] interview – discharge**	F: Δ = -4.9 [-4.5 ; -5.3]		F: Δ = -5.7 [-4.6 ; -6.9]	
	M: Δ = -3.6 [-2.6 ; -4.6]		M: Δ = -0.6 [-3.5 ; +2.2]	
**ASA classification mean (± SD) t-Test**	F: 2.44 (± 0.69)	F: 2.15 (± 0.69)	F: 2.10 (± 0.68)	F: 1.80 (± 0.70)
	M: 2.44 (± 0.76)	M: 2.14 (± 0.79)	M: 1.78 (± 0.69)	M: 1.69 (± 0.72)
	p = 0.89	p = 0.71	p < 0.0001	p = 0.056
**Δ ASA [95% CI] interview – discharge**	F: Δ = -0.29 [-0.26 ; -0.32]		F: Δ = -0.30 [-0.24 ; -0.37]	
	M: Δ = -0.30 [-0.24 ; -0.36]		M: Δ = -0.09 [-0.24 ; +0.05]	
